# Associations of workplace violence with depression and anxiety among health-care workers: the mediating role of perceived work stress

**DOI:** 10.1080/16549716.2026.2685441

**Published:** 2026-07-30

**Authors:** Xiaohui Zhai, Zhongliang Zhou, Hongbin Fan, Guanping Liu, Yan Zhuang, Zhichao Wang

**Affiliations:** a School of Public Health, Health Science Center, Xi’an Jiaotong University, Xi’an, Shaanxi, China; b School of Public Policy and Administration, Xi’an Jiaotong University, Xi’an, Shaanxi, China

**Keywords:** Workplace violence, health—care workers, mental health, mediation, cross-sectional study

## Abstract

**Background:**

Workplace violence (WPV) is a pervasive occupational hazard in health-care settings and has increasingly been recognized as a major threat to health-care workers’ mental health. Previous studies have consistently shown that WPV is associated with a range of adverse psychological outcomes, including depression and anxiety. However, despite the growing body of evidence documenting these associations, the underlying mechanisms through which WPV affects mental health remain insufficiently understood.

**Objectives:**

This study aims to examine the associations between WPV and depression and anxiety among health-care workers and to assess whether perceived work stress mediates these associations.

**Methods:**

A cross-sectional survey was conducted among health-care workers in Xi’an, China, using a multistage stratified cluster sampling design. Logistic regression models were used to assess associations between WPV and mental health outcomes, and the Karlson–Holm–Breen method was applied to assess the mediating role of perceived work stress.

**Results:**

A total of 39,773 participants were included in the final analysis, of whom 46.18% reported experiencing WPV. WPV was associated with higher odds of depression (OR = 3.79, 95% CI: 1.76–8.15) and anxiety (OR = 3.81, 95% CI: 2.11–6.90). Perceived work stress partially mediated these relationships, accounting for 18.90% of the association with depression and 15.63% with anxiety.

**Conclusions:**

WPV was significantly associated with depression and anxiety among health-care workers and perceived work stress may represent a potential pathway linking WPV to these outcomes. Interventions should focus on preventing WPV and strengthening mental health support for affected staff.

## Paper Context

- **Main findings:** Exposure to workplace violence was strongly associated with higher odds of depression and anxiety among health-care workers, and these associations were partly explained by higher perceived work stress.
- **Added knowledge:** This study provides large-scale evidence from China that perceived work stress may represent an important pathway linking workplace violence to poor mental health.
- **Global health impact for policy and action:** The findings underscore the need for integrated policies that both prevent workplace violence in health-care settings and address work-related stress through organizational and mental health support interventions.

## Background

Workplace violence (WPV) has emerged as a significant public health and occupational safety concern worldwide, particularly in health-care settings where staff frequently interact with patients and their families. WPV encompasses physical assaults, verbal abuse, bullying, harassment, and other forms of aggressive behavior experienced by employees in the course of their work [1]. According to the World Health Organization, health-care workers face a disproportionately high risk of violence, with estimates indicating that up to 62% have experienced some form of violence during their careers [2]. Consistent with these estimates, systematic reviews and large-scale surveys across different regions report prevalence rates of approximately one-third to more than two-thirds among health professionals [3,4]. The high prevalence of WPV imposes a substantial economic burden on health-care systems. For instance, a report by the American Hospital Association estimated that hospitals in the United States incurred approximately $18.27 billion annually in costs related to WPV, including prevention expenditures and postevent costs, such as treatment of injured staff, staffing losses, legal processes, and infrastructure repair [5].

Beyond its economic and systemic impact, WPV is widely recognized as a significant health risk for health-care workers. Within the social determinants of health framework, it constitutes a harmful occupational exposure that affects their health and well-being. One of the most immediate consequences of WPV is physical injury, surveillance data indicate that health-care workers face a nearly fivefold higher risk of WPV-related injuries than other occupational groups [6]. Physical assaults in clinical practice often result in acute injuries, such as bruises [7], fractures [8], and musculoskeletal disorders [9], as well as physical pain [10]. Even in the absence of physical contact, repeated exposure to verbal abuse and threats has been linked to physiological stress responses, such as sleep disturbances [11], gastrointestinal symptoms [12], and increased blood pressure [13]. Notably, these physical harms often co-occur with or may contribute to mental health conditions, which further compromise the overall well-being of health-care workers.

A growing body of evidence indicates that WPV is strongly associated with adverse mental health outcomes among health-care workers, particularly depression and anxiety. For example, a study conducted in China demonstrated that health-care workers exposed to verbal abuse had an 82% higher likelihood of depression and an 83% higher likelihood of anxiety, while those exposed to physical violence had a 27% higher likelihood of depression and a 32% higher likelihood of anxiety compared with their unexposed peers [14]. Similar findings have been documented in the United States, Lebanon, and Italy [15–17]. Longitudinal studies further suggest that the psychological consequences of WPV extend beyond the immediate aftermath, with symptoms of post-traumatic stress disorder (PTSD), anxiety, and depression persisting for several months following violent incidents [18].

However, existing studies have often focused on specific professional groups or single institutions, which may constrain the generalizability of findings. These limitations are especially relevant in complex health-care systems, such as China, where substantial variation in service demand, patient volume, and workforce capacity across different levels of health-care institutions may shape both exposure to WPV and its mental health consequences. Yet, evidence examining these associations across diverse institutional settings remains limited. More importantly, the mechanisms through which WPV influences mental health remain insufficiently understood.

The stress process framework provides a useful theoretical perspective for understanding how WPV may influence mental health. It conceptualizes stress as a dynamic process in which external stressors influence psychological outcomes through intervening processes rather than acting directly [19]. Within this framework, WPV operates as a primary stressor that disrupts individuals’ sense of safety, control, and professional stability. The concept of stress proliferation further suggests that primary stressors may generate additional forms of stress, thereby extending their impact on psychological well-being [20]. Accordingly, perceived work stress may represent a form of secondary stress or subjective distress, potentially serving as an important mechanism linking WPV to mental health outcomes. Specifically, exposure to WPV may be associated with higher levels of perceived work stress, reflecting individuals’ psychological responses to repeated or threatening interpersonal encounters. This accumulated stress may, in turn, increase vulnerability to depression and anxiety.

Consistent with this theoretical perspective, empirical studies have shown that health-care workers exposed to verbal abuse, threats, or physical assaults report higher levels of stress than those without such experiences [11,18]. This is especially concerning given that health-care professionals already operate under substantial occupational stress due to long working hours, heavy workloads, and occupational risks, such as infectious disease exposure [21–23]. Moreover, a large body of research has demonstrated that elevated stress is strongly associated with depressive and anxiety symptoms among health-care workers [24–26]. Recent studies have further demonstrated that perceived stress partially mediates the relationship between WPV and outcomes, such as burnout, job satisfaction, and quality of care [27–29]. Related research on workplace mistreatment in organizational settings, such as bullying and incivility, has also considered the mediating role of psychological factors [30]. Despite these advances, research that examines the mediating role of perceived work stress in the association between WPV and depression and anxiety remains limited.

**Figure 1.**
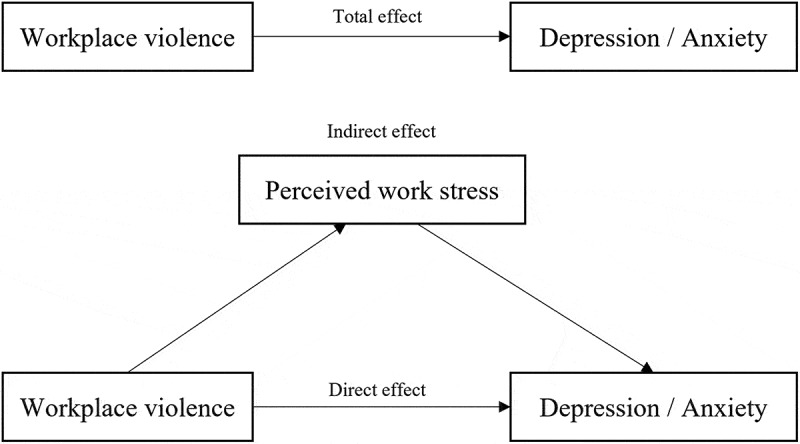
Conceptual framework of the potential mediating role of perceived work stress in the associations between workplace violence and depression and anxiety among health-care workers.

The present study aims to investigate the associations of WPV with depression and anxiety among health-care workers. Drawing on a large sample, this study further examines how perceived work stress mediates these associations, thereby clarifying the pathways through which WPV contributes to adverse mental health outcomes. The conceptual framework underlying the proposed mediation model is presented in Figure 1. The findings are expected to inform organizational strategies to reduce stressors and strengthen mental health support, which are crucial for promoting the well-being and retention of health-care workers.

## Methods

### Study design and participants

This cross-sectional study was conducted among health-care workers in Xi’an, China, covering a wide range of health-care institutions, including hospitals and primary health-care facilities. A multistage stratified cluster sampling design was used. In the first stage, nine districts in Xi’an were selected. In the second stage, health-care institutions within these districts were stratified by institution type (hospitals and primary health-care facilities), and institutions were selected using probability proportional to size (PPS) based on the number of health-care workers in each institution. All health-care workers within the selected institutions were invited to participate in the survey. The response rates were 78.27% in hospitals and 87.79% in primary health-care institutions. Eligible participants included physicians, nurses, administrative personnel, and other clinical or public health staff who were actively employed and working at the selected institutions during the study period. Participants were excluded if they were not currently employed (e.g. on long-term leave or retired), provided incomplete responses on key study variables, or failed data quality checks (e.g. implausibly short response times or inconsistent answers). After applying these criteria, 39,773 participants were included in the final analysis.

Data were collected using a structured questionnaire adapted from the physician survey of the Seventh National Health Service Survey of China. To ensure clarity, feasibility, and contextual relevance, a pilot study was conducted among 780 health-care workers drawn from institutions comparable to those included in the main survey. Feedback from the pilot study was used to refine item wording, improve response options, and optimize the structure of the questionnaire. These refinements helped enhance the comprehensibility and acceptability of the survey instrument prior to the main data collection. The final questionnaire consisted of seven sections: basic demographic information, health status and service utilization, internet-based health care, work and personal characteristics, workplace attitudes, practice environment, and perceived changes.

Data were collected online between 7 November and 8 December 2023, using the REDCap platform. Several measures were taken to ensure data quality. The online survey required completion of key items before submission, thereby reducing missing data. Logical checks were applied to identify inconsistent responses, and questionnaires with duplicate or implausible entries were excluded prior to analysis. In addition, response time was monitored to identify questionnaires completed in excessively short or long durations, which were reviewed and excluded where appropriate. All data were stored on a secure server with restricted access, and only anonymized datasets were used for statistical analyses.

This study was approved by the Xi’an Jiaotong University Biomedical Ethics Committee (approval number XJTUAE2646). Electronic informed consent was obtained from all participants. All data were handled confidentially and anonymized prior to analysis to protect participants’ privacy. Participation was voluntary, and respondents had the right to withdraw at any time.

## Measures

### Workplace violence

WPV was assessed by asking participants whether they had experienced any of the following behaviors from patients during their professional practice: hostile or negative communication, verbal abuse, physical assault, or other types of aggressive or violent acts. Participants indicated whether they had ever experienced each type of behavior, and those who reported at least one of these behaviors were classified as having experienced WPV (coded as 1), while those who reported none were coded as 0. In addition, the number of WPV types experienced was calculated by summing the four types of violent behaviors reported by each participant (range: 0–4) and was used to examine the dose–response associations between WPV exposure and mental health outcomes.

### Depression and anxiety

Depression and anxiety were each assessed based on participants’ self-reported history of a clinical diagnosis of the condition. Participants were asked whether they had ever been diagnosed with depression or anxiety by a licensed physician. Those reporting a prior diagnosis were classified as having depression or anxiety.

### Perceived work stress

Perceived work stress was assessed using a single-item measure: ‘Overall, I feel a high level of work-related stress.’ Responses were rated on a 6-point Likert scale ranging from 1 (‘strongly disagree’) to 6 (‘strongly agree’), with higher scores indicating higher levels of perceived work stress.

### Covariates

To adjust for potential confounding factors, sociodemographic and work-related characteristics were included as covariates in the analyses. Sociodemographic variables comprised gender (female, male), age (continuous), marital status (single, married), and educational attainment (associate degree or below, bachelor’s degree, master’s degree, doctoral degree). Work-related variables included occupation (physician, nurse, public health professional, allied health professional, other), professional title (no title, junior, mid-level, senior), hospital type (tertiary, secondary, primary, private), employment status (contract, permanent), and managerial position (yes, no), tenure at the current institution (continuous), monthly income (continuous), and weekly working hours (continuous).

## Statistical analysis

Descriptive statistics were used to summarize participants’ sociodemographic and work-related characteristics. Continuous variables were presented as medians and interquartile ranges (IQRs), while categorical variables were presented as frequencies and percentages. Differences between participants with and without WPV exposure were assessed using the Mann–Whitney *U* test for continuous variables and the $\chi^{2}$ test for categorical variables.

Multivariable logistic regression models were used to examine the associations between WPV exposure and depression and anxiety, respectively. For each outcome, three models were estimated sequentially: Model 1 included WPV only; Model 2 further adjusted for sociodemographic and work-related characteristics; Model 3 additionally adjusted for perceived work stress. Results were reported as odds ratios (ORs) with 95% confidence intervals (CIs). Multicollinearity was assessed using variance inflation factors (VIFs), with all values below 5, indicating no evidence of problematic multicollinearity. To assess dose–response relationships, WPV exposure was treated as a continuous variable representing the number of WPV types experienced, and models were re-estimated accordingly. All analyses accounted for the complex sampling design by incorporating sampling weights, which were calculated as the inverse of the probability of selection under the multistage stratified probability PPS design, with further adjustment for non-response.

To examine whether perceived work stress mediates the association between WPV and mental health outcomes (depression and anxiety), we applied the Karlson–Holm–Breen (KHB) decomposition method based on logistic regression models given the binary outcomes [31]. This approach partitions the total effect of WPV into direct and indirect effects through the mediator, while accounting for covariates. Compared with traditional mediation approaches, KHB is particularly advantageous in nonlinear models, such as logistic regression, as it provides unbiased estimates of mediation effects even when the outcome is binary. All sociodemographic and work-related covariates included in the main regression models were controlled for in the decomposition.

Sensitivity analyses were conducted to examine the robustness of the findings. Specifically, inverse probability of treatment weighting (IPTW) was applied to evaluate whether potential sample selection bias or differences in sample distribution across groups influenced the observed associations.

All analyses were conducted using Stata version 17.0, with a two-sided *p* < 0.05 considered statistically significant.

## Results

### Participant characteristics

Table 1 summarizes the characteristics of the health-care workers by WPV exposure. A total of 39,773 health-care workers were included in the analysis, of whom 18,368 (46.18%) reported exposure to WPV. The sample was primarily composed of nurses (36.63%) and physicians (25.35%). The majority were female (77.99%), with a median age of 34 years. Most participants were married (74.96%), held a bachelor’s degree (62.01%) and worked in tertiary hospitals (57.01%). Nearly half (45.63%) held junior professional titles. About one-quarter (26.8%) reported holding a managerial role, and 37.39% held permanent positions. The median tenure at the current institution was 7 years, with a median monthly income of 6000 yuan. Participants typically worked 42 h/week and reported a median work stress score of 4.

**Table 1.** Characteristics of the study sample.

Characteristics	Total (*N* = 39,773)	No WPV Exposure (*n* = 21,405)	WPV Exposure (*n* = 18,368)	*P*
	*n* (%)/Median (IQR)	*n* (%)/Median (IQR)	*n* (%)/Median (IQR)	
Gender				<0.001
Female	30,991 (77.99)	16,858 (78.87)	14,133 (76.97)	
Male	8744 (22.01)	4516 (21.13)	4228 (23.03)	
Age	34 (29–41)	35 (29–42)	33 (29–40)	<0.001
Marital status				<0.001
Single	9949 (25.04)	4917 (23.01)	5032 (27.41)	
Married	29,780 (74.96)	16,453 (76.99)	13,327 (72.59)	
Education				<0.001
Associate degree or below	8590 (21.60)	5654 (26.41)	2936 (15.98)	
Bachelor’s degree	24,665 (62.01)	12,940 (60.45)	11,725 (63.83)	
Master’s degree	5711 (14.36)	2446 (11.43)	3265 (17.78)	
Doctoral degree	807 (2.03)	365 (1.71)	442 (2.41)	
Occupation				<0.001
Physician	10,072 (25.35)	4354 (20.38)	5718 (31.14)	
Nurse	14,554 (36.63)	7390 (34.58)	7164 (39.01)	
Public health	2722 (6.85)	1861 (8.71)	861 (4.69)	
Allied health professional	6133 (15.44)	3553 (16.63)	2580 (14.05)	
Other	6250 (15.73)	4210 (19.70)	2040 (11.11)	
Professional titles				<0.001
No title	4025 (10.13)	2736 (12.80)	1289 (7.02)	
Junior	18,134 (45.63)	9907 (46.34)	8227 (44.81)	
Mid-level	12,959 (32.61)	6576 (30.76)	6383 (34.76)	
Senior	4622 (11.63)	2160 (10.10)	2462 (13.41)	
Hospital type				<0.001
Tertiary hospitals	22,662 (57.01)	11,351 (53.07)	11,311 (61.59)	
Secondary hospitals	7586 (19.08)	4084 (19.09)	3502 (19.07)	
Primary hospitals	7174 (18.05)	4903 (22.92)	2271 (12.37)	
Private hospitals	2331 (5.86)	1051 (4.91)	1280 (6.97)	
Employment status				<0.001
Contract employee	23,133 (62.61)	12,759 (63.56)	10,374 (61.48)	
Permanent employee	13,813 (37.39)	7314 (36.44)	6499 (38.52)	
Managerial position				<0.001
No	29,112 (73.20)	15,338 (71.66)	13,774 (74.99)	
Yes	10,661 (26.80)	6067 (28.34)	4594 (25.01)	
Tenure at the current institution	7 (3–13)	7 (3–14)	7 (3–13)	<0.001
Monthly income	6000 (4000–8500)	6000 (4000–8000)	6000 (4500–9000)	<0.001
Weekly working hours	42 (40–50)	40 (40–50)	45 (40–50)	<0.001
Perceived work stress	4 (3–5)	4 (3–5)	4 (4–5)	<0.001

Notes: ‘Single’ includes those who were never married, divorced, or widowed. ‘Other’ includes administrative, managerial, teaching, and research staff not directly involved in clinical or public health services. Monthly income was reported in Chinese Yuan (CNY).

Significant differences were observed between health-care workers with and without WPV exposure across all characteristics (*p* < 0.001). Those exposed to WPV were slightly younger, more likely to be single, and had higher educational attainment. Physicians and nurses were more likely to report WPV exposure than other occupational categories, and WPV was more prevalent among male participants, those with mid-level professional titles, and those employed in tertiary hospitals. Participants exposed to WPV reported higher monthly income, slightly longer weekly working hours, and higher perceived work stress levels. They were also more likely to be permanent employees but less likely to hold managerial positions.

### Prevalence and distribution of WPV

Table 2 presents the prevalence of each type of WPV and the distribution of the number of WPV types experienced among health-care workers. Hostile or negative communication was the most common form of WPV (40.58%), followed by verbal abuse (10.95%) and other aggressive or violent acts (7.42%). Physical assault had the lowest prevalence (1.38%). Regarding the number of WPV types experienced, 53.93% of participants reported no exposure to WPV, whereas 34.17% experienced one type of WPV. In addition, 11.89% of participants reported exposure to two or more types of WPV.

**Table 2.** Prevalence of each type of WPV and distribution of the number of WPV types experienced among health-care workers.

Variable	*n*	%
Panel A. Types of WPV		
Hostile or negative communication	16,186	40.58
Verbal abuse	4366	10.95
Physical assault	551	1.38
Other aggressive or violent acts	2959	7.42
Panel B. Number of WPV types experienced		
0	21,510	53.93
1	13,629	34.17
2	3868	9.70
3	807	2.02
4	69	0.17

### Associations of WPV with depression and anxiety

The associations of WPV with depression and anxiety are presented in Table 3. In the fully adjusted models (Model 3), WPV exposure was associated with substantially higher odds of both depression (OR = 3.79, 95% CI: 1.76–8.15) and anxiety (OR = 3.81, 95% CI: 2.11–6.90) compared with no exposure.

**Table 3.** Associations between WPV and depression and anxiety among health-care workers.

Variables	Depression, OR (95% CI)			Anxiety, OR (95% CI)		
	Model 1	Model 2	Model 3	Model 1	Model 2	Model 3
WPV	5.70** (2.00, 16.27)	4.50*** (2.16, 9.37)	3.79** (1.76, 8.15)	4.52*** (2.53, 8.07)	4.38*** (2.40, 7.98)	3.81*** (2.11, 6.90)
Gender (reference = female)						
Male		1.07 (0.64, 1.76)	0.96 (0.56, 1.66)		1.23 (0.69, 2.19)	1.05 (0.63, 1.76)
Age		0.91* (0.84, 0.99)	0.93* (0.88, 0.99)		0.95** (0.91, 0.99)	0.96* (0.92, 0.99)
Marital status (reference = single)						
Married		0.65 (0.40, 1.05)	0.53* (0.31, 0.92)		1.01 (0.45, 2.29)	0.94 (0.40, 2.21)
Education (reference = associate degree or below)						
Bachelor’s degree		0.87 (0.51, 1.48)	0.71 (0.40, 1.28)		0.34** (0.16, 0.74)	0.31** (0.14, 0.69)
Master’s degree		0.36 (0.07, 1.87)	0.56 (0.15, 2.11)		0.16* (0.04, 0.66)	0.20* (0.05, 0.82)
Doctoral degree		–	–		0.06 (0.003, 1.27)	0.12 (0.01, 2.15)
Occupation (reference = physician)						
Nurse		1.27 (0.68, 2.39)	1.33 (0.64, 2.78)		0.83 (0.28, 2.49)	0.83 (0.29, 2.39)
Public health		0.81 (0.45, 1.45)	0.73 (0.40, 1.32)		0.83 (0.34, 2.03)	0.76 (0.31, 1.88)
Allied health professional		0.63 (0.32, 1.24)	0.54 (0.24, 1.18)		1.24 (0.43, 3.55)	1.17 (0.42, 3.25)
Other		1.38 (0.68, 2.77)	1.48 (0.77, 2.87)		0.91 (0.44, 1.90)	0.91 (0.42, 1.95)
Professional titles (reference = no title)						
Junior		2.13 (0.96, 4.70)	2.49 (0.99, 6.28)		2.01 (0.68, 5.92)	1.93 (0.75, 5.00)
Mid-level		10.10*** (2.97, 34.41)	10.13** (2.67, 38.49)		3.30* (1.02, 10.70)	2.93 (0.94, 9.15)
Senior		3.75* (1.25, 11.26)	3.66* (1.09, 12.24)		3.37 (0.77, 14.80)	2.97 (0.72, 12.25)
Hospital type (reference = tertiary hospitals)						
Secondary hospitals		0.17* (0.04, 0.69)	0.19** (0.06, 0.61)		0.45 (0.20, 1.02)	0.42* (0.19, 0.95)
Primary hospitals		0.19* (0.05, 0.72)	0.21* (0.06, 0.71)		0.41* (0.18, 0.95)	0.40* (0.18, 0.88)
Private hospitals		0.13 (0.01, 1.21)	0.17 (0.02, 1.41)		0.19 (0.05, 0.80)	0.20* (0.05, 0.84)
Employment status (reference = contract employee)						
Permanent employee		2.04* (1.11, 3.75)	2.14* (1.15, 3.97)		4.40*** (1.99, 9.77)	4.38*** (1.92, 10.04)
Managerial position (reference = no)						
Yes		0.79 (0.53, 1.16)	0.70 (0.45, 1.11)		0.64* (0.44, 0.95)	0.62* (0.41, 0.93)
Tenure at the current institution		1.09* (1.01, 1.17)	1.07* (1.00, 1.13)		0.99 (0.96, 1.02)	0.98 (0.95, 1.01)
ln (monthly income)		0.52* (0.32, 0.86)	0.53** (0.34, 0.85)		0.73 (0.52, 1.03)	0.73 (0.52, 1.03)
Weekly working hours		1.02* (1.00, 1.03)	1.01 (1.00, 1.02)		1.01* (1.00, 1.02)	1.01 (1.00, 1.02)
Perceived work stress			1.71*** (1.37, 2.14)			1.52*** (1.21, 1.91)
Constant	0.01*** (0.01, 0.02)	18.15 (0.07, 5049.40)	1.24 (0.01, 204.44)	0.04*** (0.03, 0.06)	2.42 (0.07, 85.74)	0.56 (0.01, 30.14)

Notes: Odds ratios (ORs) and 95% confidence intervals (CIs) are reported, with CIs shown in parentheses. Model 1 = crude; Model 2 = adjusted for sociodemographic factors and work-related characteristics; Model 3 = fully adjusted. *p < 0.05; **p < 0.01; ***p < 0.001. Participants with a doctoral degree were omitted from the regression model for depression due to perfect prediction.

In addition to WPV, several factors were consistently associated with both depression and anxiety after full adjustment. Permanent employment was associated with higher odds of both depression (OR = 2.14, 95% CI: 1.15–3.97) and anxiety (OR = 4.38, 95% CI: 1.92–10.04). Higher perceived work stress was strongly associated with higher odds of both depression (OR = 1.71, 95% CI: 1.37–2.14) and anxiety (OR = 1.52, 95% CI: 1.21–1.91). Older age was associated with lower odds of both outcomes (depression: OR = 0.93, 95% CI: 0.88–0.99; anxiety: OR = 0.96, 95% CI: 0.92–0.99). Additionally, working in nontertiary hospitals was associated with lower odds of both depression and anxiety, with those in secondary (depression: OR = 0.19, 95% CI: 0.06–0.61; anxiety: OR = 0.42, 95% CI: 0.19–0.95) and primary hospitals (depression: OR = 0.21, 95% CI: 0.06–0.71; anxiety: OR = 0.40, 95% CI: 0.18–0.88) having lower odds compared to those in tertiary hospitals.

Moreover, being married was associated with lower odds of depression compared with being single (OR = 0.53, 95% CI: 0.31–0.92). Higher income was also associated with lower odds of depression (OR = 0.53, 95% CI: 0.34–0.85). In contrast, individuals with mid-level professional titles had higher odds of depression compared with those without a title (OR = 10.13, 95% CI: 2.67–38.49). Longer tenure at the current institution was also associated with higher odds of depression (OR = 1.07, 95% CI: 1.00–1.13). Individuals with a bachelor’s degree (OR = 0.31, 95% CI: 0.14–0.69) and a master’s degree (OR = 0.20, 95% CI: 0.05–0.82) had lower odds of anxiety compared with those with an associate degree or below. Working in private hospitals was associated with lower odds of anxiety (OR = 0.20, 95% CI: 0.05–0.84). Individuals holding a managerial position had lower odds of anxiety than those without such roles (OR = 0.62, 95% CI: 0.41–0.93).

As shown in Table 4, a clear dose–response relationship was observed between the number of WPV types experienced and mental health outcomes. A higher number of WPV types were associated with higher odds of depression (OR = 2.25, 95% CI: 1.57–3.23) and anxiety (OR = 2.37, 95% CI: 1.75–3.21) after adjusting for sociodemographic and work-related characteristics.

**Table 4.** Associations between the number of WPV types experienced and depression and anxiety among health-care workers.

	Depression			Anxiety		
Variables	OR	95% CI	*P*	OR	95% CI	*P*
Number of WPV	2.25	1.57–3.23	<0.001	2.37	1.75–3.21	<0.001
Gender (reference = female)						
Male	1.02	0.60–1.72	0.943	1.12	0.65–1.93	0.678
Age	0.92	0.86–0.99	0.026	0.95	0.92–0.99	0.011
Marital status (reference = single)						
Married	0.59	0.37–0.95	0.030	0.97	0.45–2.11	0.947
Education (reference = associate degree or below)						
Bachelor’s degree	0.77	0.42–1.43	0.411	0.33	0.15–0.72	0.005
Master’s degree	0.73	0.20–2.63	0.633	0.26	0.07–0.93	0.038
Doctoral degree	–	–	–	0.14	0.01–2.67	0.194
Occupation (reference = physician)						
Nurse	1.31	0.65–2.63	0.446	0.82	0.28–2.44	0.721
Public health	0.69	0.37–1.29	0.242	0.77	0.31–1.91	0.567
Allied health professional	0.53	0.25–1.13	0.100	1.18	0.41–3.36	0.757
Other	1.52	0.75–3.05	0.244	0.88	0.41–1.87	0.734
Professional titles (reference = no title)						
Junior	3.01	0.99–9.17	0.052	2.25	0.86–5.87	0.098
Mid-level	12.26	2.64–56.91	0.001	3.41	1.08–10.75	0.036
Senior	4.16	1.03–16.80	0.045	3.33	0.85–13.01	0.083
Hospital type (reference = tertiary hospitals)						
Secondary hospitals	0.20	0.06–0.63	0.006	0.49	0.22–1.11	0.086
Primary hospitals	0.22	0.07–0.75	0.015	0.47	0.21–1.05	0.066
Private hospitals	0.18	0.02–1.66	0.129	0.25	0.06–1.01	0.052
Employment status (reference = contract employee)						
Permanent employee	1.94	0.99–3.79	0.052	4.18	2.01–8.69	<0.001
Managerial position (reference = no)						
Yes	0.70	0.46–1.08	0.109	0.62	0.41–0.95	0.028
Tenure at the current institution	1.07	1.00–1.15	0.048	0.98	0.96–1.01	0.319
ln (monthly income)	0.52	0.32–0.85	0.008	0.73	0.52–1.03	0.070
Weekly working hours	1.01	1.00–1.02	0.080	1.01	1.00–1.02	0.031
Perceived work stress	1.82	1.41–2.36	<0.001	1.59	1.24–2.03	<0.001
Constant	1.13	0.01–209.71	0.964	0.39	0.01–23.77	0.652

Notes: Participants with a doctoral degree were omitted from the regression model for depression due to perfect prediction.

### The mediating role of perceived work stress in the association between WPV and mental health

Table 5 shows the mediating effect of perceived work stress on the associations between WPV and mental health outcomes. The total effect of WPV on depression was 1.27 (95% CI: 0.86–1.69, *p* < 0.001), of which 0.24 (95% CI: 0.12–0.35, *p* < 0.001) was mediated through perceived work stress, accounting for 18.90% of the total effect. Similarly, the total effect of WPV on anxiety was 1.28 (95% CI: 0.93–1.63, *p* < 0.001), with an indirect effect of 0.20 (95% CI: 0.12–0.28, *p* < 0.001) via perceived work stress, representing 15.63% of the total effect.

**Table 5.** Mediating effect of perceived work stress on the associations between exposure to WPV and depression and anxiety.

	Depression			Anxiety		
	*β* (95% CI)	*P*	Proportion Mediated (%)	*β* (95% CI)	*P*	Proportion Mediated (%)
Total effect	1.27 (0.86–1.69)	<0.001	–	1.28 (0.93–1.63)	<0.001	–
Direct effect	1.04 (0.65–1.43)	<0.001	–	1.08 (0.73–1.42)	<0.001	–
Indirect effect	0.24 (0.12–0.35)	<0.001	18.90	0.20 (0.12–0.28)	<0.001	15.63

### Sensitivity analyses

Sensitivity analyses yielded results consistent with those of the main analyses (Table 6). WPV exposure remained significantly associated with higher odds of depression (OR = 1.82, 95% CI: 1.60–2.07) and anxiety (OR = 1.97, 95% CI: 1.81–2.14), indicating that the findings were robust after accounting for potential selection bias.

**Table 6.** Associations between WPV and depression and anxiety using inverse probability weighting.

	Depression			Anxiety		
Variables	OR	95% CI	*P*	OR	95% CI	*P*
WPV	1.82	1.60–2.07	<0.001	1.97	1.81–2.14	<0.001
Gender (reference = female)						
Male	1.08	0.92–1.27	0.357	0.94	0.84–1.05	0.263
Age	0.99	0.97–1.00	0.117	0.99	0.98–1.00	0.263
Marital status (reference = single)						
Married	1.01	0.85–1.20	0.913	1.06	0.94–1.19	0.313
Education (reference = associate degree or below)						
Bachelor’s degree	1.24	1.00–1.54	0.055	1.22	1.06–1.40	0.007
Master’s degree	1.25	0.90–1.73	0.178	1.14	0.93–1.40	0.203
Doctoral degree	0.79	0.46–1.33	0.371	0.98	0.71–1.35	0.917
Occupation (reference = physician)						
Nurse	1.00	0.80–1.24	0.972	0.90	0.78–1.03	0.131
Public health	0.78	0.55–1.10	0.163	0.93	0.74–1.16	0.508
Allied health professional	0.99	0.80–1.23	0.914	0.97	0.84–1.12	0.690
Other	0.95	0.75–1.20	0.644	0.96	0.83–1.13	0.653
Professional titles (reference = no title)						
Junior	1.31	0.96–1.79	0.085	1.51	1.21–1.88	<0.001
Mid-level	1.49	1.06–2.07	0.020	2.05	1.62–2.60	<0.001
Senior	1.21	0.81–1.81	0.360	1.62	1.22–2.15	0.001
Hospital type (reference = tertiary hospitals)						
Secondary hospitals	1.06	0.90–1.25	0.498	1.04	0.94–1.17	0.436
Primary hospitals	0.90	0.70–1.16	0.415	0.85	0.72–1.00	0.046
Private hospitals	0.40	0.06–2.88	0.362	0.95	0.40–2.25	0.915
Employment status (reference = contract employee)						
Permanent employee	1.32	1.12–1.56	0.001	1.14	1.02–1.27	0.018
Managerial position (reference = no)						
Yes	0.95	0.81–1.11	0.496	0.99	0.89–1.10	0.835
Tenure at the current institution	1.03	1.02–1.04	<0.001	1.02	1.01–1.03	<0.001
ln (Monthly income)	0.85	0.79–0.92	<0.001	0.91	0.85–0.97	0.003
Weekly working hours	1.01	1.006–1.01	<0.001	1.01	1.006–1.01	<0.001
Perceived work stress	1.52	1.42–1.62	<0.001	1.52	1.46–1.58	<0.001
Constant	0.01	0.002–0.02	<0.001	0.01	0.004–0.01	<0.001

## Discussion

Nearly half of health-care workers reported experiencing WPV. Exposure to WPV was significantly associated with higher odds of depression and anxiety. Furthermore, perceived work stress partially mediated these associations, highlighting the importance of stress-related processes in linking WPV to adverse mental health outcomes. Based on a large-scale survey covering a wide range of health-care institutions, including both hospitals and primary health-care facilities, these results provide important evidence regarding the associations between WPV and mental health among health-care workers. By identifying the potential mediating role of perceived work stress, this study contributes to a more comprehensive understanding of how WPV affects mental health among health-care workers. These results are particularly relevant in health-care settings characterized by high work demands and frequent patient contact, where exposure to WPV may compound existing stressors and increase vulnerability to psychological distress.

Our study found that 46.18% of health-care workers experienced WPV, similar to findings reported in prior studies. Systematic reviews have estimated that 30–70% of health-care professionals worldwide experience WPV, most often in the form of verbal abuse [3,32]. In China, a large-scale survey reported a prevalence of 56.4% among health-care workers overall [33], while studies in high-risk departments, such as emergency care, have documented substantially higher levels, approaching 80% [34]. These variations are likely attributable to differences in study populations, health-care settings, and definitions of WPV. The persistently high prevalence of WPV highlights the importance of examining its psychological consequences and underscores the urgent need for effective strategies to reduce WPV in health-care settings.

Exposure to WPV was significantly associated with greater likelihood of depression and anxiety. This is consistent with previous research identifying WPV as a major risk factor for adverse mental health outcomes among health-care workers [35–37]. Such an association can be largely explained by the traumatic and threatening nature of WPV. As a form of negative workplace event, WPV triggers intense emotions, such as fear, anger, and helplessness [38], and may disrupt health-care workers’ sense of safety in the work environment. In addition, unlike many other job stressors, WPV directly threatens personal safety and professional dignity. When health-care workers perceive their workplace as unsafe or disrespectful, their commitment and professional identity may be undermined [39,40], which fosters feelings of alienation and emotional exhaustion and increases the risk of depression and anxiety [41,42].

Our study further showed that perceived work stress partially mediated the associations between WPV and depression and anxiety. Previous studies have reported that WPV is associated with increased work stress among health-care workers [43,44]. WPV often creates a threatening and unpredictable environment that undermines employees’ sense of safety and control, making everyday tasks more stressful. WPV also increases job demands, as health-care workers must devote additional effort to managing conflicts, maintaining professional communication, and completing incident reports and related documentation. These added demands amplify workload and perceived work stress [45]. In turn, perceived work stress is a well-established predictor of poor mental health outcomes [46]. Prolonged exposure to stress not only sustains activation of the hypothalamic–pituitary–adrenal axis, leading to physiological dysregulation [47], but also depletes emotional and cognitive resources, thereby impairing individuals’ capacity to regulate negative emotions [48]. As a result, individuals experiencing persistent work stress are more vulnerable to depression and anxiety.

These findings point to important implications for policy and practice, particularly in LMIC settings facing similar health system constraints. Reducing WPV should be prioritized as a fundamental occupational health and safety goal, requiring system-level interventions such as improved institutional security, formal reporting and accountability mechanisms, and effective patient–provider communication strategies. In addition, interventions should address the stress responses associated with WPV. This includes providing timely psychological support following violent incidents, establishing clear postincident response protocols, and fostering supportive work environments that help health-care workers cope with violence-related stress. Such approaches may help reduce the psychological burden associated with WPV and mitigate its adverse impact on mental health. Particular attention should be paid to vulnerable groups within the health-care workforce, such as those working in high-risk departments or with limited institutional resources, with interventions tailored to their specific work conditions and support needs. By integrating preventive and supportive strategies, health-care systems can more effectively safeguard the mental health of their workforce and ensure the sustainability of care delivery.

## Limitations

This study has several limitations that should be acknowledged. First, the cross-sectional design limits the ability to establish causal relationships between WPV and mental health outcomes. Although significant associations were observed, the temporal sequence cannot be determined, and causal inferences should be made with caution. Longitudinal studies are warranted to clarify causal pathways. Second, depression and anxiety were assessed through self-reported physician diagnosis rather than standardized clinical interviews or validated symptom scales. Such measures are subject to recall bias and may be influenced by mental health-care accessibility and stigma, potentially leading to underestimation of the true prevalence of depression and anxiety. Third, WPV was measured only in relation to patients, without considering other possible sources, such as relatives, colleagues, or supervisors. Fourth, despite the use of a multistage stratified sampling approach, the study was conducted in Xi’an, China, and the findings may not be generalizable to health-care workers in other regions or countries with different health-care systems and workplace environments. Finally, this study did not account for organizational-level factors, including organizational support, workplace safety policies, and reporting and feedback systems, which may have introduced residual confounding. Future research should integrate both individual- and organizational-level determinants to better understand the mechanisms linking workplace environments, violence, and mental health outcomes among health-care workers.

## Conclusion

This study demonstrated that WPV was significantly associated with higher odds of depression and anxiety among health-care workers, with perceived work stress partially mediating these associations. By identifying perceived work stress as a key pathway, the findings offer insight into the mechanisms underlying the psychological consequences of WPV. These results underscore the need to reduce violence in health-care settings and strengthen psychosocial support systems to protect health-care workers’ mental health and sustain health-care system resilience.

## Data Availability

The data that support the findings of this study are available from the corresponding author upon reasonable request.
